# Prolonged infusion of remimazolam in surgical and medical intensive care unit patients: a pilot pharmacokinetic study

**DOI:** 10.1186/s40560-025-00840-9

**Published:** 2025-12-11

**Authors:** Yuji Suzuki, Matsuyuki Doi, Yoshitaka Aoki, Hiromi Kato, Kensuke Kobayashi, Soichiro Mimuro, Takashi Mochizuki, Takahiro Yamada, Motoyasu Miura, Shinya Uchida, Yoshiki Nakajima

**Affiliations:** 1 Department of Intensive Care Medicine, Hamamatsu University School of Medicine, Hamamatsu, Japan; 2 Department of Intensive Care Medicine, Hamamatsu Medical Center, Hamamatsu, Japan; 3 Department of Hospital Pharmacy, Hamamatsu University School of Medicine, Hamamatsu, Japan; 4 Departments of Pharmacy Practice and Science, School of Pharmaceutical Sciences, University of Shizuoka, Shizuoka, Japan

**Keywords:** Remimazolam, Pharmacokinetics, Drug Clearance, Volume of distribution, Mechanical ventilation, Intensive care units, Continuous infusion, Benzodiazepines, Intra- and intersubject variability, Sedative agents

## Abstract

**Background:**

The pharmacokinetics of prolonged remimazolam infusion in patients undergoing long-term mechanical ventilation remain unclear. This study aimed to evaluate the pharmacokinetics of remimazolam administered continuously for 24 h.

**Methods:**

This open-label pharmacokinetic analysis enrolled patients requiring mechanical ventilation into two groups: the surgical group, which received remimazolam during and after surgery, and the medical ICU group, which received remimazolam in the intensive care unit (ICU). Remimazolam was administered at a fixed rate of 0.1 mg/kg/h for ≥ 24 h, and blood samples were collected at regular intervals. Plasma remimazolam concentrations were measured by tandem mass spectrometry.

**Results:**

Twenty patients (10 in each group) completed the study. The median duration of remimazolam infusion was 24.0 h in the surgical group and 102.0 h in the medical ICU group. The steady-state plasma concentrations in both the surgical and medical ICU groups exhibited modest intrasubject variability (4.46–32.73%) and moderate intersubject variability (16.45–31.71%), with all values falling within clinically acceptable intermediate ranges. The plasma remimazolam concentration at the end of infusion was 130.7 ng/mL (95% confidence interval [CI] 115.2–146.1) in the surgical group and 134.3 ng/mL (95% CI 98.7–170.0) in the medical ICU group. Noncompartmental analysis showed that the clearance was 54.3 L/h (95% CI 47.6–61.8) and 55.6 L/h (95% CI 42.8–72.1) (*P* = 0.856), while the volume of distribution at steady state was 284 L (95% CI 215–376) and 316 L (95% CI 142–707) (*P* = 0.780), with no statistically significant differences between the groups.

**Conclusions:**

In this preliminary study, both the surgical ICU group (approximately 24 h) and the medical ICU group (beyond 24 h) showed no evidence of time-dependent accumulation of plasma remimazolam, indicating a generally stable pharmacokinetic profile under the examined conditions.

*Trial registration*: In compliance with the Japanese Clinical Trials Act, the study was classified as a Specified Clinical Trial owing to the use of unapproved pharmaceuticals, which were reviewed by a certified review board (CRB) and registered in the Japan Registry of Clinical Trials (jRCTs041200076) on December 15, 2020.

**Supplementary Information:**

The online version contains supplementary material available at 10.1186/s40560-025-00840-9.

## Background

The choice of a sedative agent for patients undergoing mechanical ventilation is a critical component of intensive care, as inappropriate sedation can lead to complications, such as hemodynamic instability, delayed extubation, and delirium. Propofol, dexmedetomidine, and benzodiazepines are commonly used for sedation; however, each has specific limitations. Propofol carries a higher risk of hypotension than benzodiazepines and may cause propofol infusion syndrome with prolonged use, especially in children. Its lipid emulsion formulation also increases the risk of fatty acid overload and infection [1–4]. Dexmedetomidine has a slow onset of action and can cause adverse effects, such as bradycardia and cardiac conduction disturbances [5]. Benzodiazepines, such as midazolam and lorazepam, have prolonged half-lives, which can lead to delayed recovery of neuromotor function compared with propofol. In addition, some benzodiazepines produce pharmacologically active metabolites. Because benzodiazepines increase the risk of delirium, the Clinical Practice Guidelines for the Prevention and Management of Pain, Agitation/Sedation, Delirium, Immobility, and Sleep Disruption recommend minimizing their use in critically ill patients [6].

Remimazolam is a novel benzodiazepine that is rapidly metabolized by hepatic carboxylesterase 1, yielding pharmacologically inactive metabolites. This metabolic profile enables rapid clearance, even after prolonged administration, making remimazolam a promising candidate for sedation in intensive care settings, where precise titration of sedation depth is required [7]. Based on these characteristics, the ONO-2745-04 trial was conducted in Japan to evaluate the efficacy and safety of remimazolam for sedation of patients undergoing mechanical ventilation. The trial was terminated early because of unexpectedly high plasma concentrations observed in some patients receiving continuous infusion beyond 24 h, raising concerns about overdose and prolonged sedation. Although plasma concentrations of remimazolam during a 24-h infusion have been reported in recent years [8], its pharmacokinetic (PK) profile beyond 24 h remains insufficiently characterized. Further investigations are warranted to clarify the time course of drug levels during extended administration.

We hypothesized that, under fixed-rate continuous infusion conditions, plasma drug concentrations would remain stable in both inter- and intrapatient comparisons, even during infusion periods exceeding 24 h, similar to those observed with a 24-h infusion. This study investigated whether prolonged remimazolam infusion combined with adjunctive sedatives could prevent excessive plasma drug accumulation while maintaining adequate sedation in mechanically ventilated patients.

## Methods

### Study design and ethics

This prospective, open-label, single-center pilot study was conducted in accordance with the Declaration of Helsinki and approved by the Institutional Review Board of Hamamatsu University School of Medicine (Approval No. C016-2020) on December 1, 2020. In compliance with the Japanese Clinical Trials Act, this study was classified as a Specified Clinical Trial owing to the use of unapproved pharmaceuticals, which were reviewed by a certified review board and registered in the Japan Registry of Clinical Trials (jRCTs041200076) on December 15, 2020. The Clinical Research Quality Assurance Division performed clinical audits at the Clinical Research Center of Hamamatsu University School of Medicine. This study was conducted between January 2021 and April 2024 at the Department of Anesthesiology and Intensive Care Medicine, Hamamatsu University School of Medicine. The study was reported in accordance with the CONSORT 2010 extension for pilot and feasibility trials and was adapted to a non-randomized single-arm design.

### Study population

Participants were enrolled based on two distinct protocols, and written informed consent was obtained from either the participant or a legally authorized representative. Because this was an exploratory study, no formal sample size calculations were performed.

#### Surgical group

Patients undergoing elective surgery requiring ≥ 24 h of sedation and mechanical ventilation were eligible. Inclusion criteria included continuous intravenous sedation and an anticipated intensive care unit (ICU) stay of ≥ 24 h. The exclusion criteria were age < 20 years, hypersensitivity to study-related drugs, acute angle-closure glaucoma, myasthenia gravis, neurological disorders, history of seizures, asthma, pregnancy, cognitive or psychiatric disorders, hepatic dysfunction, and communication difficulties. Ten participants were enrolled in this group.

#### Medical ICU group

Patients who did not undergo elective or emergency surgery and required ≥ 24 h of sedation and mechanical ventilation in the ICU were included. Patients with burns were included in this study. The exclusion criteria were the same as those used for the surgical group. Twenty participants were enrolled in this group.

### Study procedures

**Fig. 1:**
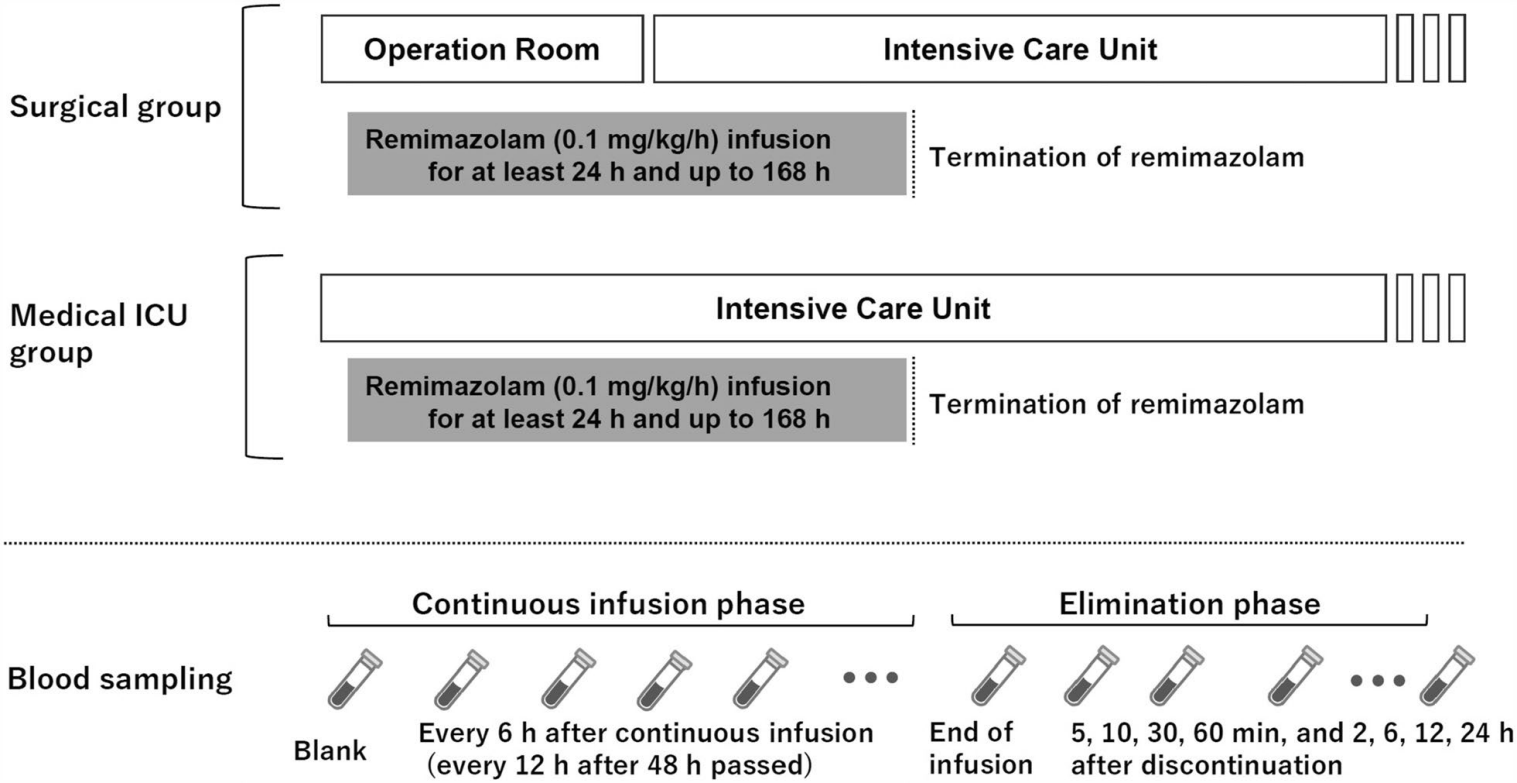
Schematic illustration of the study protocol. Remimazolam administration schedule in the surgical and medical ICU groups. Both groups received a continuous remimazolam infusion at a rate of 0.1 mg/kg/h. The minimum infusion duration was 24 h, and administration was discontinued either at the end of sedation management or at 168 h, whichever occurred first

An overview of the study protocol is shown in Fig. 1. In both the surgical and medical ICU groups, propofol was administered to induce loss of consciousness, followed by rocuronium administration to facilitate tracheal intubation. Subsequently, remimazolam (diluted with 0.9% sodium chloride solution to a concentration of 1 mg/mL) was continuously infused at a fixed rate of 0.1 mg/kg/h, calculated according to actual body weight, via a peripheral vein, peripherally inserted central catheter, or central venous catheter. In the ONO-2745-04 trial, patients ventilated postoperatively were assigned to starting infusion rates of 0.1, 0.25, or 0.5 mg/kg/h, with subsequent adjustments based on sedation depth. Among these, 0.1 mg/kg/h represented the lowest starting rate, and a time-dependent increase in plasma remimazolam concentrations was reported in some patients receiving infusions longer than 24 h. Based on these findings and ethical considerations, the present study adopted a fixed infusion rate of 0.1 mg/kg/h to minimize oversedation risk and exclude confounding effects of dose adjustments, thereby enabling a clear assessment of potential time-dependent accumulation during prolonged administration. Remimazolam infusion was continued for ≥ 24 h and was discontinued either at the end of sedation or after a maximum of 168 h. The concomitant use of other anesthetics (sevoflurane and desflurane), sedatives (propofol and dexmedetomidine), and analgesics (e.g., fentanyl and remifentanil) was permitted, except for benzodiazepines. The depth of sedation required for general anesthesia or intensive care was assessed using either an electroencephalographic monitor (BSM-3000 series; Nihon Kohden, Japan) or the Richmond Agitation–Sedation Scale. To maintain the target level of sedation, the doses of the anesthetic or sedative agents were adjusted as needed.

### Data collection

Baseline characteristics, including age, sex, height, weight, comorbidities, surgical indication, medication history, and preoperative laboratory data, were obtained from clinical records and patient interviews. Hemodynamic parameters, sedation scores, adverse events, and PK blood samples were collected according to predefined protocols. In addition, the dose and duration of the investigational drug, as well as the dose and duration of all administered anesthetics, analgesics, and sedatives, were recorded during mechanical ventilation. The length of the ICU stay was also recorded.

### PK sampling and analysis

#### Blood sample collection

An arterial catheter was placed in all participants for PK sampling. Blood (2 mL) was collected in ethylenediaminetetraacetic acid-containing tubes (Venoject EDTA-2 K^®^, Terumo, Japan).

Blood samples were collected according to the same schedule in both the surgical and medical ICU groups. A blank sample was obtained before initiating continuous remimazolam infusion. Post-initiation samples were collected at 6, 12, 18, 24, 30, 36, 42, 48, 60, 72, 84, 96, 108, 120, 132, 144, 156, and 168 h after the start of the infusion, provided that remimazolam was continuously administered at each corresponding timepoint (minimum duration: 24 h). Blood samples were collected immediately before the discontinuation of remimazolam infusion and at 5, 10, 30, and 60 min and 2, 6, 12, and 24 h after discontinuation.

#### Plasma concentration measurement

The collected blood samples were immediately stored at 4 °C and then centrifuged at 3000 × *g* for 10 min within 12 h, after which the plasma was separated. The separated plasma samples were stored at − 80 °C and analyzed within 1 month of collection.

The plasma concentrations of remimazolam and its metabolite, CNS 7054, were measured using a validated high-performance liquid chromatography–tandem mass spectrometry method. The samples were processed by protein precipitation, and d4-remimazolam was used as an internal standard. The assays for remimazolam and CNS 7054 were validated over the concentration ranges of 0.5–200 ng/mL and 5–600 ng/mL, respectively, with intra- and interassay accuracy and relative standard deviations (SD) of less than 15%. Samples that exceeded the upper limit of quantification were diluted with a blank matrix before analysis.

All measurements of plasma drug and metabolite concentrations were performed by two pharmacists (TM and TY), anonymized to patient information, and conducted in accordance with the US Food and Drug Administration “Bioanalytical Method Validation Guidance for Industry” (2018) [9].

### Primary outcome

The primary outcome of this study was the intra- and intersubject variability in plasma remimazolam concentration during continuous infusion. To evaluate the stability of plasma concentrations, all plasma samples obtained during the infusion period, which were assumed to represent a steady state, were included in the analysis of both the surgical and medical ICU groups.

### Assessment of variability in plasma drug concentrations

The intrasubject coefficient of variation (CV_intra_) was calculated for each subject using all plasma concentration data obtained during the infusion period, which were assumed to reflect steady-state conditions. The coefficient of variation (CV = SD/mean × 100) was used to quantify the variability. A CV_intra_ < 15% was considered very low, and < 30% was considered low-to-moderate variability based on regulatory and literature standards [10].

Intersubject variability (CV_inter_) was assessed differently between the surgical and medical ICU groups. In the surgical group, CV_inter_ was calculated at each steady-state timepoint (6, 12, 18, and 24 h) using the mean and SD of the plasma concentrations across the subjects. In contrast, the medical ICU group was evaluated using a time block approach, whereby the infusion period was divided into consecutive 24-h intervals (e.g., 0–24, 24–48, and 48–72 h), and CV_inter_ was calculated based on the average plasma concentration within each block. CV_inter_ was stratified as follows: ≤ 10% (very low), < 25% (moderate), and > 40% (high), with the intermediate range (25–40%) considered moderate-to-high variability, according to published PK conventions [11]. CV_inter_ was calculated only when ≥ 4 patients had available data within the time interval.

In the surgical ICU group, the infusion period was approximately 24 h and blood-sampling times were well-aligned; therefore, CV_inter_ was calculated at prespecified steady-state timepoints every 6 h. In contrast, medical ICU patients received prolonged, uninterrupted infusions well beyond 24 h, typically over multiple days. Because routine clinical procedures, diagnostic tests, and nursing care often caused minor shifts in sampling times, we evaluated intersubject variability using 24-h time blocks to mitigate potential bias related to timing discrepancies. In addition, we predefined an outlier criterion and planned a sensitivity analysis in which CV_inter_ was re-estimated after excluding observations meeting this criterion to assess the robustness of the variability estimates.

To minimize the influence of apparent outliers, a sensitivity analysis was conducted by excluding predefined atypical cases, defined as patients exhibiting plasma concentrations exceeding 1.5 times the interquartile range (IQR) at three or more timepoints during or after the infusion period. The calculation of CV_inter_ for each 24-h block was performed only when data from at least four patients were available within the corresponding interval.

A steady-state assessment was performed using the data set after excluding outliers. Steady state was defined as CV_intra_ < 30%, a threshold commonly used in regulatory evaluations of PK variability and supported by previous clinical PK literature [10].

### PK analysis

PK analysis was performed at the termination of continuous remimazolam infusion using a noncompartmental approach. The PK parameters of remimazolam and CNS 7054 were estimated assuming the complete metabolic conversion of remimazolam to CNS 7054. Because a direct proof of complete conversion was not feasible, this assumption was made for analytical convenience in the present study. PK analysis was performed by two pharmacokineticists (SU and MM) independently of drug administration and plasma concentration measurements. All analyses were conducted using Phoenix WinNonlin software (version 8.4.0; Certara, NJ, USA).

### Safety, tolerability, and interim analysis

Safety assessments included physical examinations, vital signs, and laboratory tests. Blood pressure (systolic, diastolic, and mean arterial pressures) was continuously monitored using an arterial catheter. Heart rate was continuously recorded using electrocardiography. Oxygen saturation was monitored using pulse oximetry. Routine hematology, clinical chemistry, and coagulation parameters were also assessed. Adverse events were documented according to their nature, severity, duration, and outcomes. Hemodynamic and respiratory management during remimazolam administration was conducted by the attending anesthesiologist in the operating room and intensivists in the ICU. The use of other sedatives and vasoactive agents was permitted; however, the study protocol was discontinued in the event of life-threatening hypotension or hypoxia, including persistent hypotension unresponsive to vasoactive agents (mean blood pressure ≤ 50 mmHg), cardiac arrest, or hypoxia requiring extracorporeal membrane oxygenation.

Predefined stopping criteria for the entire study were also established to address drug-related safety concerns. If a serious adverse event leading to death or severe permanent sequelae occurred and was judged to be related to remimazolam administration, the trial was halted and reviewed by an independent safety board. Interim analyses were planned after every 10 participants completed both remimazolam administration and plasma sampling to confirm the feasibility of the PK measurements and validate the assay performance.

### Statistical analysis

Continuous demographic and clinical variables were expressed as mean (SD) or median [IQR] according to their distribution and were compared between groups using the Welch *t* test or the Mann–Whitney *U* test, as appropriate, based on the Shapiro–Wilk test for normality. Categorical variables are summarized as counts (%) and compared using the chi-squared test or Fisher exact test. Plasma concentrations at steady state were summarized as arithmetic means with SDs, because concentrations were expected to be approximately normally distributed under steady-state conditions. PK parameters obtained from the noncompartmental analysis were log-transformed prior to analysis, and between-group comparisons were performed using the Welch *t* test on the log-transformed data. Geometric mean ratios with 95% confidence intervals (CIs) were calculated using a back transformation. All statistical analyses were performed using JMP for Windows (version 14.2.0, SAS Institute Inc., Cary, NC, USA).

## Results

### Demographics

This study, conducted between January 2021 and April 2024, enrolled 10 patients in the surgical group and 13 patients in the medical ICU group. Of the 13 patients in the medical ICU group, three were excluded after providing informed consent: two because tracheal intubation was ultimately avoided, and one due to clinical deterioration leading to death before the study intervention. The surgical group consisted of 10 postoperative patients, including four who underwent esophageal cancer resection and six who underwent oral or laryngeal surgery. The medical ICU group comprised 10 critically ill patients with respiratory failure, including those with bacterial pneumonia (*n* = 2), flail chest (*n* = 1), interstitial pneumonia (*n* = 1), pulmonary contusion (*n* = 1), pulmonary congestion due to heart failure (*n* = 1), pulmonary congestion secondary to oliguria from acute kidney injury (*n* = 1), cervical spinal cord injury (*n* = 1), extensive burns (*n* = 1), and tetanus (*n* = 1). Although we initially aimed to enroll 20 patients in the medical ICU group, recruitment was halted at 10 patients because of challenges, such as the COVID-19 pandemic. All the enrolled patients received remimazolam as planned and completed the study. Demographic data are summarized in Table 1.

**Table 1:** . Patient characteristics

	Surgical group	Medical ICU group
Age (year)	69.6 (9.0)	63.3 (19.9)
Sex (male/female)	8/2	6/4
Height (cm)	159.9 (8.6)	164.1 (11.2)
Weight (kg)	64.5 (8.4)	66.3 (12.4)
BMI (kg/m^2^)	25.5 (5.1)	24.5 (3.3)
Hematocrit (%)	37.8 (2.7)	35.2 (7.1)
Hemoglobin (g/dL)	12.7 (0.9)	11.6 (2.4)
Platelets count (× 10^9^/µL)	257 (79)	154 (90)
Plasma fibrinogen (mg/dL)	433 (87)	521 (212)
Fibrin/fibrinogen degradation products (µg/mL)	1.25 [1.0–2.12]	3.05 [1.87–20.4]
PT-INR	0.97 (0.10)	1.10 (0.18)
Soluble fibrin (µg/mL)	6.6 (2.9)	19.7 (13.1)
BUN (mg/dL)	16.3 (5.6)	26.0 [16.3–38.4]
Creatinine (mg/dL)	0.83 (0.25)	0.99 [0.80–1.16]
Total bilirubin (mg/dL)	0.65 (0.15)	0.87 (0.34)
LDH (IU/L)	191 (40)	333 (173)
AST (IU/L)	18.0 (4.0)	34 [30.5–64.5]
ALT (IU/L)	13.1 (5.3)	28.6 (11.4)
ALP (IU/L)	89.1 (22.6)	62.3 (16.8)
GGT (IU/L)	35.4 (21.0)	25.1 (9.4)
ChE (IU/L)	238 (99)	225 (53)
Total protein (g/dL)	6.41 (0.42)	5.19 (0.85)
Albumin (g/dL)	3.77 (0.43)	2.88 (0.42)
C-reactive protein (mg/dL)	0.65 [0.17–1.24]	8.32 (6.38)
Total remimazolam dose (mg)	154 [138–170]	675 [474–889]
Remimazolam infusion duration (h)	24 [24–24.3]	102 [81.7–145.5]
Anesthesia duration (min)	654 [619–690]	N/A
Artificial respiration duration after remimazolam termination(h)	3.0 [1.0–24.2]	63.1 [3.5–234.1]
Length of ICU stay after remimazolam termination (h)	39.9 [38.8–41.3]	194.2 [99.5–407.7]

Data are presented as mean (standard deviation), median [interquartile range], or number (percentage) of patients, as appropriate

BMI, body mass index; PT-INR, prothrombin time—international normalized ratio; BUN, blood urea nitrogen; LDH, lactate dehydrogenase; AST, aspartate aminotransferase; ALT, alanine aminotransferase; ALP, alkaline phosphatase; GGT, gamma-glutamyl transferase; ChE, choline esterase; N/A, not applicable; ICU, intensive care unit

### Sedation protocols and duration of remimazolam infusion

All patients in both the surgical and medical ICU groups received a continuous infusion of remimazolam in combination with other sedatives (details are shown in Supplementary Table S1). In the surgical group, nine patients (90%) received sevoflurane and one patient (10%) received desflurane during surgery; no other inhalational or intravenous anesthetics were coadministered. In the postoperative ICU period, propofol was administered to all patients, and dexmedetomidine was coadministered to eight patients (80%). In the medical ICU group, propofol and dexmedetomidine were administered to six patients (60%) and nine patients (90%), respectively. The median duration of remimazolam infusion was 24.0 h [24.0–24.3] (range 24.0–25.0) in the surgical group and 102.0 h [81.7–145.5] (range 42.0–159.0) in the medical ICU group. In both groups, no patients exhibited an unresponsive comatose state during ICU management, except when deep sedation was deliberately induced.

### Safety analysis

Among the patients receiving continuous remimazolam infusion, one episode of severe hypotension (mean arterial pressure < 50 mmHg) occurred in a patient with sepsis in the medical ICU group shortly after sedation initiation. The patient’s condition improved rapidly after administration of norepinephrine and arginine vasopressin. No other adverse events were reported.

### Variability and pharmacokinetics of remimazolam and CNS 7054

#### Surgical group

**Fig. 2:**
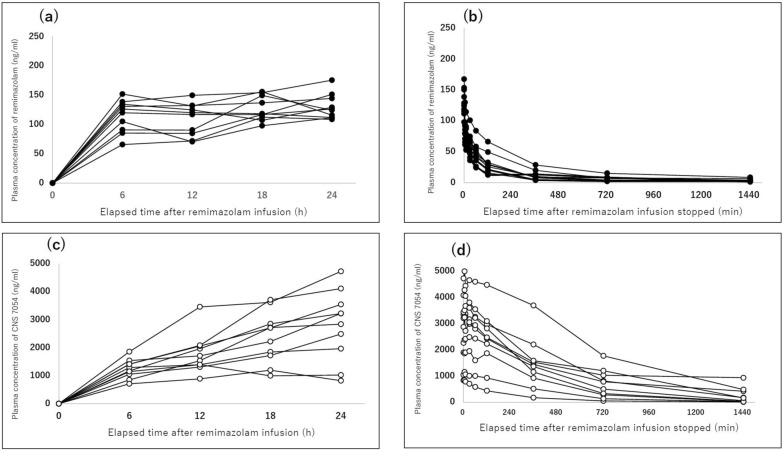
Plasma concentrations of remimazolam and its metabolite CNS 7054 in the surgical group. **a** Plasma remimazolam concentrations during continuous infusion. **b** Plasma remimazolam concentrations after termination of infusion. **c** Plasma CNS 7054 concentrations during remimazolam infusion. **d** Plasma CNS 7054 concentrations after termination of infusion. Filled circles represent remimazolam concentrations and open circles represent CNS 7054 concentrations in each individual case

All blood samples were collected as scheduled, yielding 140 plasma concentration measurements for PK modeling. The time courses of remimazolam and CNS 7054 plasma concentrations during and after infusion are shown in Fig. 2. The CV_intra_ ranged from 4.46% to 27.12% among the 10 participants. The mean CV_intra_ was 14.74% (95% CI 9.05–20.42), indicating modest within-subject variability in plasma drug concentrations approximately 24 h. All patients demonstrated CV_intra_ values below 30%, confirming that the study population could be reasonably considered to have achieved steady-state conditions. Individual CV_intra_ values as well as the mean and SD of plasma concentrations per patient are summarized in Table 2. After discontinuation of the infusion, remimazolam concentrations declined rapidly, reflecting limited persistence in the plasma and a rapid elimination profile.

**Table 2:** Intrasubject variability in plasma concentrations (CV_intra_) approximately 24 h in the surgical group

Subject ID	Mean (SD) remimazolam concentration (ng/mL)	CV_intra_ (%)	Range (min–max)
A-01	103.37 (21.42)	20.72	85.38–126.38
A-02	89.69 (24.32)	27.12	65.71–112.44
A-03	126.38 (16.77)	13.27	116.93–151.44
A-04	136.19 (6.07)	4.46	130.95–144.46
A-05	154.69 (15.46)	9.99	138.74–175.64
A-06	113.85 (28.64)	25.15	90.49–149.57
A-07	119.27 (5.88)	4.93	112.02–126.30
A-08	124.03 (11.87)	9.57	107.25–134.63
A-09	138.89 (18.64)	13.42	115.91–155.97
A-10	96.24 (18.03)	18.73	70.51–111.47
Mean [95% CI]		14.74 [9.05–20.42]	

CV_intra,_ coefficient of variation within each subject across 6–24 h; SD, standard deviation; CI, confidence interval

Each subject’s CV_intra_ was calculated using all available plasma concentrations during the infusion period. Mean (SD) values are presented as references. The arithmetic means and 95% CI of CV_intra_ are shown in the final row

CV_intra_ < 15% was considered very low, and < 30% was considered low-to-moderate variability

CV_inter_ ranged from 16.45% to 25.43% across the timepoints. The mean CV_inter_ approximately 24 h was 20.53% (95% CI 12.99–28.07), indicating moderate intersubject variability. The detailed summary statistics are presented in Table 3. In contrast, the plasma concentration of CNS 7054 increased during the infusion period and declined more slowly following the termination of remimazolam administration, indicating a slower elimination process than that of remimazolam (Fig. 2).

**Table 3:** Intersubject variability in plasma concentrations (CV_inter_) of remimazolam approximately 24 h in the surgical group

Timepoint	Mean (SD) remimazolam concentration (ng/mL)	CV_inter_ (%)	n
6 h	114.98 (27.31)	23.75	10
12 h	109.40 (27.82)	25.43	10
18 h	126.68 (20.89)	16.49	10
24 h	129.98 (21.38)	16.45	10
Mean [95% CI]		20.53 [12.99–28.07]	

CV_inter,_ coefficient of variation within each timepoint across all subjects; SD, standard deviation; CI, confidence interval

Mean (SD) values are presented as references. The arithmetic mean and 95% CI of CV_inter_ are shown in the final row. CV_inter_ was stratified as follows: ≤ 10% (very low), < 25% (moderate), and > 40% (high), with the intermediate range (25–40%) considered moderate-to-high variability

The mean plasma concentration of remimazolam immediately prior to the end of infusion was 130.7 ng/mL (95% CI 115.2–146.1). In the non-compartmental analysis, the clearance (CL) of remimazolam was 54.3 L/h (95% CI 47.6–61.8), and the steady-state distribution volume (V_ss_) was 284 L (95% CI 215–376) (Table 4).

**Table 4:** Noncompartmental analysis parameter estimates (surgical group)

Remimazolam	Geometric mean (95% CI)	Arithmetic mean (95% CI)
C_eoi_ (ng/mL)	129.1 (114.7–145.2)	130.7 (115.2–146.1)
AUC_0–τ_ (h·ng/mL)	2779 (2444–3161)	2820 (2455–3185)
AUC_0–inf_ (h·ng/mL)	2862 (2491–3289)	2911 (2504–3318)
MRT_0–τ_ (h)	3.64 (3.09–4.29)	3.72 (3.11–4.34)
MRT_0–inf_ (h)	5.24 (3.98–6.91)	5.69 (3.52–7.87)
t_1/2_ (h)	12.94 (8.31–20.15)	15.57 (7.64–23.49)
CL (L/h)	54.3 (47.6–61.8)	55.1 (47.4–62.9)
V_SS_ (L)	284 (215–376)	307 (203–412)
CNS 7054	Geometric mean (95% CI)	Arithmetic mean (95% CI)
C_eoi_ (ng/mL)	2440 (1617–3681)	2766 (1866–3666)
AUC_0–τ_(h·ng/mL)	59,715 (41,742–85,427)	66,073 (45,287–86,858)
AUC_0–inf_ (h·ng/mL)	63,388 (43,004–93,435)	71,284 (47,571–94,997)
AUC_0–inf_ ratio	22.1 (14.7–33.4)	25.2 (16.7–33.8)

CI, confidence interval; C_eoi_, concentration at end of infusion; AUC_0–τ_, area under plasma concentration–time curve from time 0 extrapolated to τ; AUC_0–inf_, area under the curve from time 0 extrapolated to infinity; MRT, mean residence time; t_1/2_, elimination half-life; CL, elimination clearance; V_ss_, distributed volume at steady state; AUC_0–inf_ ratio, the ratio of AUC_0–inf_ of CNS 7054 to that of remimazolam

#### Medical ICU group

**Fig. 3:**
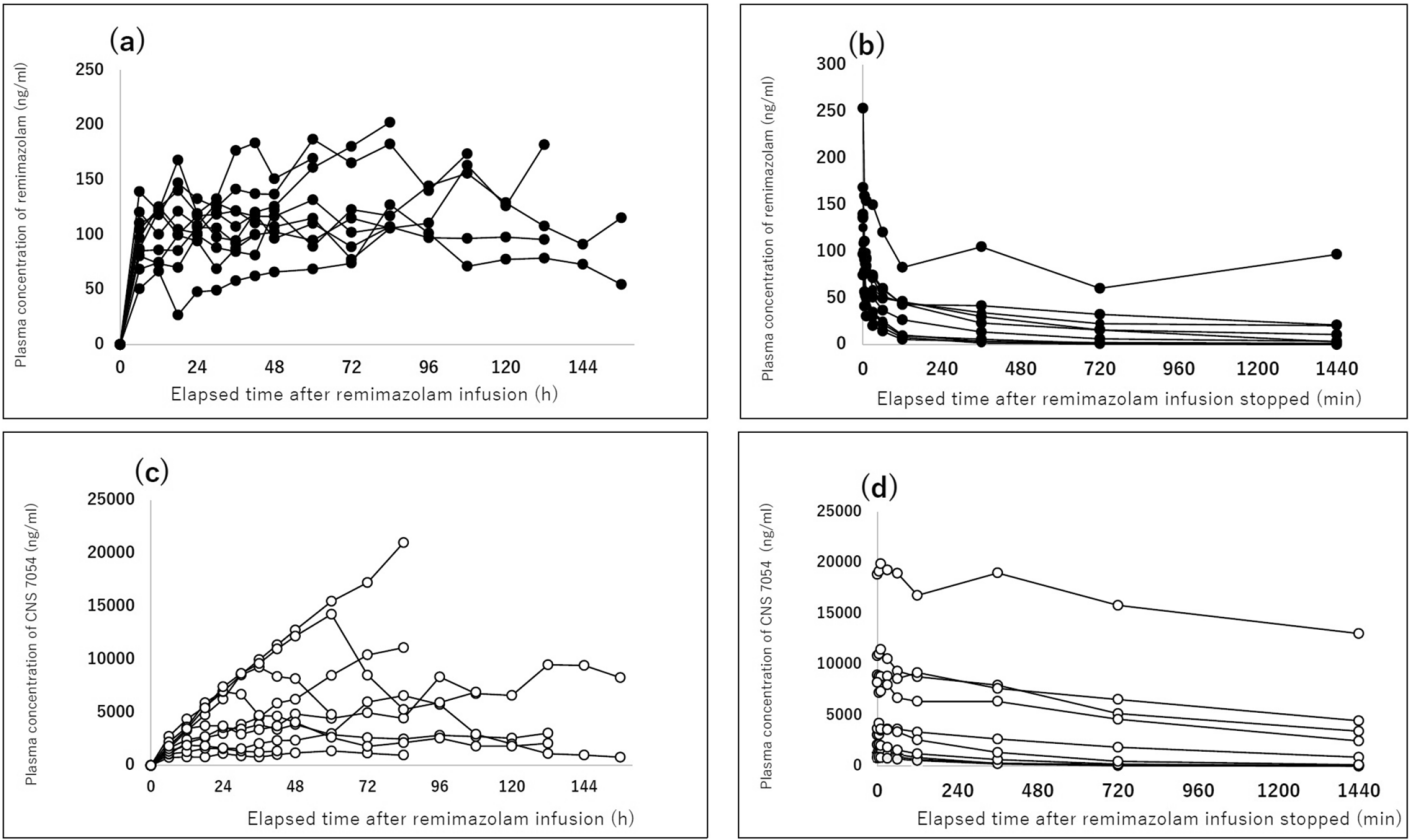
Plasma concentrations of remimazolam and its metabolite CNS 7054 in the medical ICU group. **a** Plasma remimazolam concentrations during continuous infusion. **b** Plasma remimazolam concentrations after termination of infusion. **c** Plasma CNS 7054 concentrations during remimazolam infusion. **d** Plasma CNS 7054 concentrations after termination of infusion. Filled circles represent remimazolam concentrations and open circles represent CNS 7054 concentrations for each individual case

All blood samples were collected as scheduled, resulting in 226 plasma concentration measurements that were incorporated into the PK model. The time courses of remimazolam and CNS 7054 plasma concentrations during and after infusion are shown in Fig. 3.

CV_intra_ during the infusion period (6–156 h) ranged from 10.12% to 32.78%, with a mean of 18.97% (95% CI 13.48–24.46%). Although the mean exceeded the commonly referenced threshold of 15%, most CV_intra_ values were below 30%. To verify the steady-state conditions, two outlier cases (B05 and B07) were detected based on the predefined criteria. After exclusion, all CV_intra_ values were below 30%, with a mean of 16.08% (95% CI 11.76–20.41%), supporting the conclusion that the study population was reasonably in a steady state. Mean plasma concentrations varied across individuals (range 68.03–146.10 ng/mL), but within-subject fluctuation remained limited in most cases. A summary of the individual CV_intra_ values along with the mean concentrations, SDs, and 95% CIs is presented in Table 5.

**Table 5:** Intrasubject variability (CV_intra_) in plasma remimazolam concentrations during the steady-state period in the medical ICU group

Subject ID	Mean (SD) remimazolam concentration (ng/mL)	CV_intra_ (%)	Range
B-01	107.25 (11.11)	10.35	89.08–125.54
B-02	92.11 (15.40)	16.72	68.63–115.15
B-03	110.95 (12.36)	11.14	95.75–131.97
B-04	94.76 (9.59)	10.12	85.60–106.73
B-05	68.03 (22.30)	32.78	27.00–127.38
B-06	104.46 (25.14)	24.07	70.22–155.94
B-07	134.55 (37.94)	28.20	87.23–202.47
B-08	121.23 (25.17)	20.76	77.63–182.03
B-09	144.77 (27.47)	18.97	104.54–183.69
B-10	146.10 (24.18)	16.55	110.68–186.97
Mean [95% CI]		18.97 [13.48–24.46]	

CV_intra,_ coefficient of variation within each subject across 6–156 h; SD, standard deviation; CI, confidence interval

Each subject’s CV_intra_ was calculated using all available plasma concentrations during the infusion period. Mean (SD) values are presented as references. The arithmetic means and 95% CI of CV_intra_ are shown in the final row

CV_intra_ < 15% was considered very low, and < 30% was considered low-to-moderate variability

The CV_inter_ of remimazolam plasma concentrations was assessed within consecutive 24-h time blocks throughout the infusion period. CV_inter_ ranged from 24.83% to 31.17%, with the highest variability observed during the 48–72 h interval. Across all time blocks, mean plasma concentrations remained stable. All CV_inter_ values fell within the intermediate range (25–40%), indicating consistent moderate-to-high intersubject variability (Table 6).

**Table 6:** Interindividual variability (CV_inter_) in plasma concentrations of remimazolam by 24-h time blocks in the medical ICU group

Time Block	Mean (SD) remimazolam concentration (ng/mL)	CV_inter_	n
6–24 h	101.81 (25.28)	24.83	10
24–48 h	110.65 (28.82)	26.04	10
48–72 h	117.88 (36.75)	31.17	9
72–96 h	136.66 (38.62)	28.26	6
96–120 h	114.83 (34.64)	30.16	4

CV, coefficient of variation; SD, standard deviation

The CV_inter_ values represent the percentage coefficient of variation across the individual means for each time block. Mean (SD) values are shown as reference values. CV_inter_ was calculated only when data from ≥ 4 patients were available. CV_inter_ was stratified as follows: ≤ 10% (very low), < 25% (moderate), and > 40% (high), with the intermediate range (25–40%) considered moderate-to-high variability

The mean plasma concentration of remimazolam at the end of infusion was 134.3 ng/mL (95% CI 98.7–170.0). After discontinuation, the plasma concentrations of both remimazolam and CNS 7054 rapidly declined in most patients. In contrast to the patients in the surgical group, one subject exhibited a gradual increase in plasma CNS 7054 concentration during the continuous infusion period. This patient also showed delayed elimination of both remimazolam and CNS 7054 following the cessation of infusion. These atypical PK patterns were observed in the same patient who received mechanical ventilation for sepsis and continuous renal replacement therapy (CRRT) for acute kidney injury. However, the association between impaired drug clearance and CRRT remains unclear.

In the noncompartmental analysis, the CL of remimazolam was 55.6 L/h (95% CI 42.8–72.1), and V_ss_ was 316 L (95% CI 142–707) (Table 7).

**Table 7:** Noncompartmental analysis parameter estimates (medical ICU group)

Remimazolam	Geometric mean (95% CI)	Arithmetic mean (95% CI)
C_eoi_ (ng/mL)	127.3 (100.1–161.9)	134.3 (98.7–170.0)
AUC_0–τ_(h·ng/mL)	11,465 (8199–16,032)	12,436 (9231–15,641)
AUC_0–inf_ (h·ng/mL)	11,816 (8370–16,682)	12,872 (9481–16,263)
MRT_0–τ_(h)	4.37 (1.98–9.64)	6.26 (3.30–9.21)
MRT_0–inf_ (h)	5.70 (2.59–12.54)	8.84 (3.16–14.52)
t_1/2_ (h)	10.09 (6.19–16.46)	12.28 (6.82–17.74)
CL (L/h)	55.6 (42.8–72.1)	59.2 (42.2–76.2)
V_SS_ (L)	316 (142–707)	477 (212–742)
CNS 7054	Geometric mean (95% CI)	Arithmetic mean (95% CI)
C_eoi_ (ng/mL)	3672 (1706–7905)	5910 (1750–10,070)
AUC_0–τ_(h·ng/mL)	428,158 (205,599–891,632)	605,421 (297,482–913,361)
AUC_0–inf_ (h·ng/mL)	461,167 (211,091–1,008,000)	699,760 (269,533–1,130,000)
AUC_0–inf_ ratio	39.0 (23.0–66.2)	48.7 (25.3–72.2)

CI, confidence interval; C_eoi_, concentration at end of infusion; AUC_0-τ_, area under plasma concentration–time curve from time 0 extrapolated to τ; AUC0_-inf_, area under the curve from time 0 extrapolated to infinity; MRT, mean residence time; t_1/2_, elimination half-life; CL, elimination clearance; V_ss_, distributed volume at steady state; AUC_0-inf_ ratio, the ratio of AUC_0-inf_ of CNS 7054 to that of remimazolam

#### Comparison of remimazolam pharmacokinetics in the surgical and medical ICU groups

The area under the concentration–time curve during a dosing interval (AUC_0–τ_) was 2779 (95% CI 2444–3161) h·ng/mL in the surgical group and 11,465 (95% CI 8199–16,033) h·ng/mL in the medical ICU group, with the latter being significantly higher (*P* < 0.001). In contrast, the concentration at the end of infusion was 130.7 (95% CI 115.2–146.1) ng/mL in the surgical group and 134.3 (95% CI 98.7–170.0) ng/mL in the medical ICU group, showing no significant difference (*P* = 0.834). The elimination half-life (t_1/2_) was 12.94 h (95% CI 8.31–20.15) in the surgical group and 10.09 h (95% CI 6.19–16.46) in the medical ICU group (*P* = 0.405). CL was 54.3 (95% CI 47.6–61.8) L/h in the surgical group and 55.6 (95% CI 42.8–72.1) L/h in the medical ICU group (*P* = 0.856). V_ss_ was 284 L (95% CI 215–376 L) in the surgical group and 316 L (95% CI 142–707 L) in the medical ICU group (*P* = 0.780). None of these parameters, except AUC_0–τ_, showed significant differences between the groups. (Supplementary Table S2).

### Sensitivity analysis

To minimize the influence of atypical outliers, a predefined exclusion criterion was applied: any subject exhibiting plasma concentrations exceeding 1.5 times the IQR at three or more timepoints during or after the infusion period was considered an outlier. Based on this definition, no patient in the surgical group met the exclusion criteria; however, two patients (B-05 and B-07) from the medical ICU group were identified as outliers and were excluded from the sensitivity analysis (Supplementary Figure S1). In the primary analysis, including all 10 patients, the CV_inter_ values across nearly all time intervals ranged from 25% to 40%, indicating that the intersubject variability in plasma remimazolam concentrations remained within a clinically acceptable range. In the sensitivity analysis, excluding two potential outlier cases (B-05 and B-07), the CV_inter_ values decreased at all the timepoints. After excluding outlier subjects, CV_inter_ remained at approximately 25% (Table 8).

**Table 8:** Interindividual variability (CV_inter_) of remimazolam plasma concentrations across 24-h time blocks during continuous infusion

time block	Mean (SD) remimazolam concentration (ng/mL)	CV_inter_ (%)	n
6–24 h	105.72 (17.94)	16.97	8
24–48 h	115.83 (23.56)	20.34	8
48–72 h	124.34 (33.86)	27.23	7
72–96 h	119.44 (22.97)	19.23	6
96–120 h	139.67 (31.59)	22.62	4

^*^excluding predefined outlier cases B-05 and B-07

CV, coefficient of variation; SD, standard deviation

Mean (SD) values are shown for each block. CV_inter_ was calculated only when ≥ 4 subjects had available data within the time interval. CV_inter_ was stratified as follows: ≤ 10% (very low), < 25% (moderate), and > 40% (high), with the intermediate range (25–40%) considered moderate-to-high variability

Among the two outlier patients excluded from the sensitivity analysis, one was a patient undergoing CRRT (B-07) who exhibited delayed CL of remimazolam and a marked increase in plasma CNS 7054 concentrations. Another patient had extensive burn injuries (B-05) (Fig. 3).

### Supplemental analysis

In a previous study [8], a one-compartment pharmacokinetic analysis was performed using plasma concentration data collected during a 24-h continuous infusion of remimazolam and up to 4 h after the infusion ended. The 0.1 mg/kg/h continuous infusion group in that study had dosing conditions similar to those of the surgical group in the present study. Therefore, although the plasma concentration collection window differed (4 h in the previous study vs 6 h in the present study), we conducted a supplementary analysis for reference to facilitate a comparison between the two studies. In this analysis, PK parameters were estimated using a one-compartment model based on plasma concentrations measured up to 6 h after the end of remimazolam infusion in the surgical group. The estimated t_₁/₂_ was 46.33 min (median [IQR], 37.88–59.86). The area under the plasma concentration–time curve extrapolated to infinity (AUC₀_–inf_) was 155.34 µg·min/mL (median [IQR], 150.90–170.63). CL was 15.58 mL/min/kg (median [IQR], 14.08–16.29), and the volume of distribution (V_d_) was 1011.94 mL (median [IQR], 884.19–1347.11). The full set of PK parameters is shown in Supplementary Table S3.

### Exploratory subgroup analysis within the surgical cohort (esophageal vs oral/laryngeal surgery)

As an exploratory analysis within the surgical cohort, we compared patients who underwent esophageal surgery (*n* = 4) and those who underwent oral/laryngeal surgery (*n* = 6). The baseline characteristics, intraoperative and postoperative day 1 laboratory variables, and noncompartmental PK parameters of remimazolam were analyzed. Continuous variables are expressed as median (range) and were compared using the Mann–Whitney *U* test, whereas categorical variables were compared using Fisher exact test. PK parameters (concentration at end of infusion [C_eoi_], AUC_₀–τ_, t_1/2_, CL, and V_ss_) were summarized as arithmetic mean (95% CI) because of the small sample size (*n* = 4–6 per group), which could exaggerate interindividual variability after log transformation. Between-group comparisons of PK parameters were performed using the Student *t* test.

Ten patients who underwent surgery were included in the analysis. The baseline, postoperative, and PK parameters of the esophageal surgery group were compared with those of the oral/laryngeal surgery group (Supplementary Tables S4–6). Baseline demographic and clinical characteristics were generally comparable between the groups, except that the esophageal surgery group had higher lactate dehydrogenase levels (230.5 vs 162.5 IU/L, *P* = 0.038), and the oral/laryngeal group had longer mechanical ventilation after remimazolam termination (0.85 vs 22.7 h, *P* = 0.014) (Supplementary Table S4). On postoperative day 1, the esophageal surgery group exhibited higher aspartate aminotransferase (51.5 vs 21.0 IU/L, *P* = 0.038) and serum albumin (3.30 vs 2.45 g/dL, *P* = 0.014) levels, but lower fibrinogen levels (311 vs 385 mg/dL, *P* = 0.019) than the oral/laryngeal surgery group (Supplementary Table S5). In the PK analysis, the esophageal surgery group had a lower AUC_₀–τ_ (2389 vs 3107 h·ng/mL, *P* = 0.017) and higher CL (63.8 vs 49.4 L/h, *P* = 0.029), while other parameters, including C_eoi_, t_₁/₂_, and V_ss_, did not differ significantly between groups (Supplementary Table S6).

## Discussion

To our knowledge, this is the first study to quantitatively evaluate both intra- and intersubject variability in plasma remimazolam concentrations during continuous infusion in postoperative and critically ill ICU patients, including infusions approximately 24 h (surgical ICU) and beyond 24 h (medical ICU).

The novelty of this study lies in its approach, which extends beyond evaluating sedation efficacy and safety to providing a quantitative characterization of the concentration–time profile of remimazolam, as well as a detailed analysis of its PK stability and variability during prolonged infusion. Previous in vitro studies have demonstrated that remimazolam does not significantly alter hepatic metabolic capacity or carboxylesterase-1 enzyme expression, even after 5 days of exposure [12]. Consistent with these findings, our in vivo data revealed no substantial reduction in clearance or evidence of drug accumulation, indicating stable pharmacokinetics during prolonged infusion. Plasma concentrations remained within the moderate range in most patients, and marked outliers were rare. However, a small number of patients exhibited unexpectedly elevated concentrations, potentially due to hepatic or renal dysfunction, systemic inflammation, or fluid loss. These findings are clinically relevant and should be considered in future dose planning. Overall, the rapid attainment of steady-state concentrations and absence of notable accumulation support the PK stability of remimazolam, even during extended administration.

Previous studies have reported the CL of remimazolam to be approximately 68.4 L/h (1.14 L/min) [13] and 66.6 L/h (1.11 L/min) [14], although these estimates were based on short-duration infusions. In the present study, the noncompartmental analysis yielded slightly lower CL values (54.3 L/h or 55.6 L/h). The estimated V_ss_ was 284 L and 316 L in the surgical and medical ICU groups, respectively, both greater than the previously reported 34.8 L by Antonik et al. [15]. This difference is likely explained by the distinct dosing design and PK modeling: Antonik et al. [15] applied a three-compartment model to single-bolus data in healthy volunteers, whereas the present study evaluated prolonged infusions in ICU patients using non-compartmental analysis. Continuous infusion allows for progressive redistribution to deeper compartments, leading to a larger apparent V_ss_. Nevertheless, no significant differences in CL or V_ss_ were observed between the surgical and medical ICU groups, suggesting limited time-dependent accumulation during prolonged administration. These findings support the stable PK profile of remimazolam under various clinical conditions.

In the ONO-2745-04 trial that included postoperative, mechanically ventilated patients, a time-dependent increase in plasma remimazolam concentration was suggested only in a subset of patients receiving continuous infusion beyond 24 h, and not as a uniform phenomenon across all participants. In contrast, in our medical ICU group—treated with a non-adjustable fixed infusion rate of 0.1 mg/kg/h—we observed no time-dependent increase (i.e., no progressive accumulation) in plasma concentrations during prolonged administration. This discrepancy may reflect differences in dosing design (adjustable rates vs fixed rate), patient populations and pathophysiology (postoperative vs medical ICU), and distribution conditions, such as fluid balance, serum albumin, and hemodynamics. Taken together with the absence of between-group differences in CL and V_ss_ in this study, these findings suggest that remimazolam maintains a stable PK profile during prolonged infusion under the present clinical conditions.

In addition to the primary analysis, we conducted a supplementary PK analysis using plasma concentrations up to 6 h after the end of the infusion, enabling a comparison with a previous report that employed a 4-h postinfusion sampling window. An extension to 6 h was allowed to obtain a greater number of terminal data points, reducing the proportion of extrapolated AUC0-inf and thereby improving the robustness of the estimates. In this analysis, CL was broadly consistent with the results of a previous study, suggesting a similar elimination capacity. In contrast, this study yielded a shorter t₁_/_₂ and a smaller V_d_, while λz (equivalent to k₁₀ in the one-compartment model) also differed substantially. These discrepancies may be attributable to differences in the observation window, dosing regimen, or patient population; however, the present study design and data set did not allow definitive conclusions regarding their origin. Therefore, we provide these supplementary results primarily to enhance transparency and comparability with prior literature rather than to propose mechanistic explanations. A detailed summary of PK parameters is presented in Supplementary Table S3.

Within the surgical cohort, we performed an exploratory subgroup analysis comparing patients undergoing esophageal vs oral/laryngeal surgery to generate hypotheses regarding procedure-related influences on pharmacokinetics. Although both groups had comparable total doses and infusion durations, the oral/laryngeal surgery group exhibited higher AUC_₀–τ_ and lower CL, indicating relatively reduced systemic elimination. These findings may be attributable to differences in the intraoperative hemodynamics, hepatic blood flow, or postoperative inflammatory status associated with the surgical site. Furthermore, prolonged mechanical ventilation and lower serum albumin levels observed in the oral/laryngeal surgery group may have influenced drug distribution and metabolism. However, these changes are more likely related to perioperative management specific to free-flap reconstruction and albumin replacement therapy rather than to the surgical site itself. Given the small number of patients and considerable interindividual variability, these results should be regarded as hypothesis-generating. Further studies involving larger and more homogeneous surgical populations are warranted to clarify whether surgical site-related physiological factors affect remimazolam pharmacokinetics.

Taken together, this study provides the first data on the temporal PK stability and variability of remimazolam during continuous infusion beyond 24 h in a medical ICU cohort and approximately 24 h in a surgical cohort. These findings offer a valuable foundation for individualized dosing strategies and the development of plasma concentration monitoring protocols. The time–concentration data generated in this study may support personalized sedation management and help prevent adverse events related to drug accumulation.

Overall, our findings support and extend those of previous clinical and preclinical studies [16–19] by providing detailed PK data in real-world ICU settings. In line with previous studies that have suggested the potential safety of continuous infusion beyond 24 h, the present data offer additional scientific evidence supporting the safe and effective clinical application of remimazolam in ICU sedation.

Further studies are warranted to assess the pharmacodynamic aspects of prolonged remimazolam infusion, particularly the relationships among plasma concentration, sedation depth, and clinical outcomes. In addition, there are currently no PK studies in pediatric ICU patients. Therefore, research on this vulnerable group is urgently needed to clarify the potential role of remimazolam in pediatric sedation.

Notably, one of the two outlier cases (B-05) involved a patient undergoing CRRT. The pharmacokinetics of remimazolam during CRRT remain poorly understood; potential contributing factors include membrane properties, effluent flow rates, and alterations in protein binding. These mechanisms may explain the atypical concentration profile observed in this case. The systematic evaluation of CRRT-associated pharmacokinetics should be an important focus of future research.

This study has several limitations. This single-center study had a relatively small sample size, which limits its generalizability, particularly among patients with severe organ dysfunction or complex polypharmacy. In addition, the study focused solely on pharmacokinetics and did not evaluate pharmacodynamic correlations, such as sedation depth or clinical outcomes. Furthermore, the potential confounding effects of concomitant medications or disease severity on pharmacokinetics could not be fully accounted for. Future studies should aim to identify the target plasma concentrations associated with optimal sedation. Furthermore, the infusion rate was fixed at 0.1 mg/kg/h, and the maximum infusion duration was approximately 159 h, which limited our ability to explore pharmacokinetics under higher doses or ultra-long-term administration.

## Conclusions

Continuous infusion of remimazolam for more than 24 h was associated with low intrasubject and moderate intersubject variability, without evidence of time-dependent accumulation, indicating a stable PK profile under the studied conditions. These findings support the feasibility of prolonged administration for ICU sedation; however, the emergence of outlier profiles underscores the need for larger multicenter studies to clarify the determinants of individual variability and confirm these preliminary results.

## Supplementary Information

Supplementary Material 1. Supplementary Figure S1. Plasma concentrations of remimazolam and its metabolite CNS 7054 in the medical ICU group. **a** Plasma remimazolam concentrations during continuous infusion. **b** Plasma remimazolam concentrations after termination of infusion. **c** Plasma CNS 7054 concentrations during remimazolam infusion. **d** Plasma CNS 7054 concentrations after termination of infusion. Filled circles represent remimazolam concentrations; open circles represent CNS 7054 concentrations for each individual case. Light-tone filled and open triangles represent the remimazolam and CNS 7054 concentrations of B-05, while light-tone filled and open squares represent the remimazolam and CNS 7054 concentrations of B-07

Supplementary Material 2.

Supplementary Material 3.

Supplementary Material 4.

Supplementary Material 5.

Supplementary Material 6.

Supplementary Material 7.

## Data Availability

Data are available upon reasonable request from the corresponding author to prospective researchers.

## Abbreviations

CONSORT: Consolidated Standards of Reporting Trials
ICU: Intensive care unit
RASS: Richmond agitation–sedation scale
PK: Pharmacokinetics
SD: Standard deviation
FDA: Food and Drug Administration
CV: Coefficient of variation
CV_intra_: Intrasubject coefficient of variation
CV_inter_: Intersubject coefficient of variation
IQR: Interquartile range
CI: Confidence interval
CL: Clearance
V_ss_: Steady-state distribution volume
CRRT: Continuous renal replacement therapy
AUC_0–τ_: Area under the concentration–time curve during a dosing interval
t_1/2_: Elimination half-life
AUC_0–inf_: Area under the plasma concentration–time curve extrapolated to infinity
V_d_: Volume of distribution
