# Supplementary figures and images for: Prolonged infusion of remimazolam in surgical and medical intensive care unit patients: a pilot pharmacokinetic study
Source: J Intensive Care. 2025 Dec 11;14:4. doi: 10.1186/s40560-025-00840-9 (PMC12805780; doi:10.1186/s40560-025-00840-9)

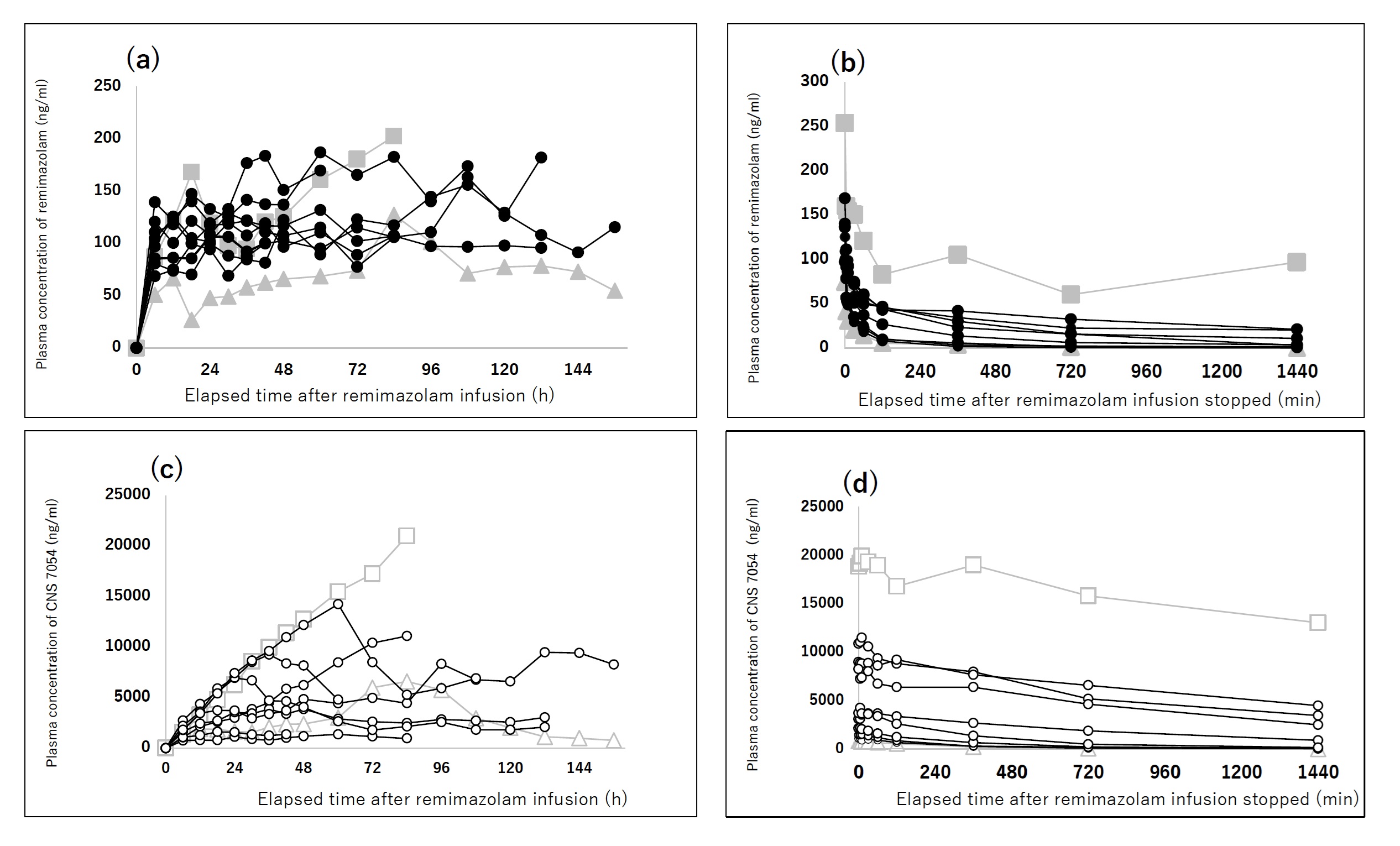

Supplement: Supplementary file 1 — Supplementary Material 1. Supplementary Figure S1. Plasma concentrations of remimazolam and its metabolite CNS 7054 in the medical ICU group. a Plasma remimazolam concentrations during continuous infusion. b Plasma remimazolam concentrations after termination of infusion. c Plasma CNS 7054 concentrations during remimazolam infusion. d Plasma CNS 7054 concentrations after termination of infusion. Filled circles represent remimazolam concentrations; open circles represent CNS 7054 concentrations for each individual case. Light-tone filled and open triangles represent the remimazolam and CNS 7054 concentrations of B-05, while light-tone filled and open squares represent the remimazolam and CNS 7054 concentrations of B-07 [file 40560_2025_840_MOESM1_ESM.jpg]
